# Maternal hypertensive disorders and subtypes of hypospadias: A Dutch case‐control study

**DOI:** 10.1111/ppe.12683

**Published:** 2020-07-13

**Authors:** Hussein Jamaladin, Iris A. L. M. van Rooij, Loes F. M. van der Zanden, Marleen M. H. J. van Gelder, Nel Roeleveld

**Affiliations:** ^1^ Department for Health Evidence Radboud Institute for Health Sciences Radboud university medical center Nijmegen The Netherlands; ^2^ Radboud REshape Innovation Center Radboud university medical center Nijmegen The Netherlands

**Keywords:** antihypertensive medication use, hypospadias, maternal hypertensive disorders, pregnancy complications; preeclampsia, subtypes of hypospadias

## Abstract

**Background:**

Hypospadias is a frequently occurring congenital anomaly in male infants, in which the opening of the urethra is located along the ventral side of the penis. Although various studies attempted to identify its causes, the aetiology of the majority of hypospadias cases remains poorly understood. Maternal hypertensive disorders are believed to be associated with hypospadias, but the results of previous studies are not consistent, especially for subtypes of hypospadias.

**Objectives:**

To investigate the associations between maternal hypertensive disorders, stratified by pharmacological treatment, and the occurrence of hypospadias divided into subtypes in a large population‐based case‐control study.

**Methods:**

We included 887 hypospadias cases and 1005 male controls from the AGORA data‐ and biobank. Cases and controls were born in the periods 1975‐2016 and 1990‐2011, respectively. All data were collected in the period 2004‐2018. Maternal questionnaires were used to obtain information on hypertensive disorders during pregnancy, antihypertensive medication treatment, and potential confounders. Adjusted odds ratios (aORs) with 95% confidence intervals (CIs) for the associations between hypertensive disorders and hypospadias were estimated using logistic regression.

**Results:**

Hypertensive disorders were reported by 15.3% of the women in this study. Maternal hypertensive disorders in general, chronic hypertension, and gestational hypertension were not associated with hypospadias or its subtypes. Preeclampsia was associated with posterior hypospadias (aOR 3.09, 95% CI 1.49, 6.43), whether it was untreated (aOR 2.81, 95% CI 1.24, 6.38) or pharmacologically treated preeclampsia (aOR 4.96, 95% CI 1.08, 22.80).

**Conclusions:**

Our findings indicate that preeclampsia is associated with posterior hypospadias, irrespective of pharmacological treatment. This result supports the hypothesis of aetiological heterogeneity among the subtypes of hypospadias, with pregnancy‐related risk factors being associated with the more severe types of hypospadias.

**Synopsis**

1 **Study question**

Are maternal hypertensive disorders, stratified by pharmacological treatment, associated with the occurrence of hypospadias divided into subtypes?

2 **What's already known**

Various studies found that maternal hypertensive disorders separately or as a group were associated with hypospadias. A few studies explored these associations further by looking at antihypertensive medication use during pregnancy and/or the subtypes of hypospadias. Previous studies suggest that associations with hypospadias are dependent on the type of hypertensive disorder, maternal exposure to antihypertensive medication during pregnancy, and the subtypes of hypospadias.

3 **What this study adds**

This study explored the associations between maternal hypertensive disorders and hypospadias in detail, by stratifying both exposures and outcomes. The results indicate that preeclampsia is associated with posterior hypospadias.

## 1 BACKGROUND

Hypospadias is one of the most frequently occurring congenital anomalies in male infants.^1^, ^2^ Boys diagnosed with hypospadias have a displaced opening of the urethra on the ventral side of the penis, due to incomplete fusion of the urethral folds in the third and fourth months of gestation.^3^, ^4^, ^5^ Based on the location of the meatus, subtypes of hypospadias can be defined as anterior hypospadias (glandular, coronal, and sub‐coronal), middle hypospadias (distal penile, midshaft, and proximal penile), and posterior hypospadias (penoscrotal, scrotal, and perineal),^6^, ^7^, ^8^ similar but not equivalent to first, second, and third‐degree hypospadias and other classifications that are being used.^9^ This birth defect is usually surgically repaired in the first 2 years after birth, but may still cause complications later in life, including social and sexual problems.^10^, ^11^ Although hypospadias is associated with specific genetic mutations in a small percentage of cases, the aetiology of the majority of hypospadias cases is still unknown.^7^, ^12^

Hypertensive disorders affect approximately 5% to 10% of pregnancies.^13^ They are divided into four mutually exclusive groups: chronic hypertension, gestational hypertension, preeclampsia, and preeclampsia superimposed on chronic hypertension.^14^ Various studies suggested possible associations between hypospadias or its subtypes and maternal hypertensive disorders and/or the use of antihypertensive medication during pregnancy.^6^, ^8^, ^15^, ^16^, ^17^, ^18^, ^19^, ^20^, ^21^, ^22^, ^23^, ^24^, ^25^, ^26^ Some of these studies reported that hypertensive disorders as a group were associated with hypospadias.^18^, ^21^, ^22^, ^25^ The majority of previous studies, however, divided the hypertensive disorders into subgroups and reported that chronic hypertension,^26^ gestational hypertension,^6^, ^16^, ^19^, ^20^, ^25^ and/or preeclampsia^6^, ^8^, ^15^, ^17^, ^20^, ^24^, ^26^ were associated with hypospadias. Other studies reported that neither chronic^15^ nor gestational hypertension^26^ was associated with hypospadias. A few studies investigated these associations in more detail by looking at antihypertensive medication use during pregnancy and subtypes of hypospadias. Untreated hypertension and late treated hypertension were found to be associated with second‐ and third‐degree hypospadias in three studies,^18^, ^21^, ^22^ while these subtypes of hypospadias were associated with untreated gestational hypertension and untreated preeclampsia in another.^23^ In the latter study, early treated chronic hypertension was associated with first‐degree hypospadias.^23^ Unrelated to treatment, preeclampsia was found to be associated strongly with posterior hypospadias and only weakly with anterior and middle hypospadias.^6^

As stated above, previous studies showed rather consistent results, but suggest that associations with hypospadias are dependent on the type of hypertensive disorder, maternal exposure to antihypertensive medication during pregnancy, and subtype of hypospadias. So far, however, only one study reported on a combination of all three of these factors.^23^ Therefore, we explored the associations between the separate maternal hypertensive disorders, stratified by pharmacological treatment, and the occurrence of hypospadias including its subtypes in a large population‐based case‐control study.

## 2 METHODS

### 2.1 Case‐control selection

For this case‐control study, we used data from the AGORA (Aetiologic research into Genetic and Occupational/environmental Risk factors for Anomalies in children) data‐ and biobank at the Radboud university medical center in the Netherlands. The continuous data collection for AGORA started in December 2004. During the first hospital visit, parents of children diagnosed with or treated for a birth defect are asked to participate in the AGORA data‐ and biobank. For specific birth defects, such as hypospadias, hospital records were used to also identify older cases for retrospective data collection. Population‐based controls were recruited through random sampling from 39 municipal registries throughout the Netherlands in 2011. The participation rates were 34% among control parents and >65% among case parents. Data have been collected using parental questionnaires, by reviewing medical records, and by collecting blood or saliva from children and parents for DNA extraction. The data collection methods for AGORA were described in detail elsewhere.^27^ For this study, we included all cases with any type of hypospadias with or without other congenital malformations and controls registered in the AGORA data‐ and biobank in the period 2004‐2018. Cases were born in the period 1975‐2016 and controls in 1990‐2011. In case of two or more brothers with hypospadias, only the oldest patient was included. The control group did not contain any siblings. Multiple gestations, female children (in the control group), and mothers diagnosed with pre‐existing diabetes were excluded. The case population consisted for almost 60% of cases included in two earlier studies of our group using a different control group.^6^, ^8^

### 2.2 Exposures

All exposure information was collected using the AGORA questionnaire, among others containing questions on chronic illnesses, pregnancy‐related complications, and medication use in the three months before conception and during pregnancy, divided into gestational weeks or months (months 1‐2, months 3‐4, and months 5‐9). Our primary exposures of interest were the four mutually exclusive groups of maternal hypertensive disorders as recommended in the Report of the National High Blood Pressure Education Working Group on High Blood Pressure in Pregnancy^14^: chronic hypertension, gestational hypertension, preeclampsia, and preeclampsia superimposed on chronic hypertension. In addition, we stratified each exposure group into women who were or were not treated with antihypertensive medication for their condition, as a likely proxy for severity of all hypertensive disorders and as specific exposure during the aetiologically relevant time window for chronic hypertension. Maternal age at delivery (18‐29 years, 30‐34 years, or ≥35 years), maternal ethnicity based on country of birth and self‐reported ethnic background (European Caucasian vs other), maternal education (low (elementary school or lower vocational education), intermediate (secondary school or intermediate vocational education), or high (higher vocational education, or university)), parity (0 vs ≥1 previous births), pre‐pregnancy body mass index (BMI)(<18.5 kg/m^2^ (underweight), 18.5‐24.9 kg/m^2^ (healthy weight), 25.0‐29.9 kg/m^2^ (overweight), and ≥30.0 kg/m^2^ (obese)), fertility treatment (yes vs no), periconceptional (any time from 3 months preconception through the first 3 months of pregnancy) oral contraceptive use (yes vs no), folic acid supplement use in the recommended period (entire recommended period, only part of recommended period, or no), periconceptional alcohol use (yes vs no), periconceptional smoking (yes vs no), and family history of hypospadias (father or brother of the child with hypospadias) were assessed as potential confounders or effect modifiers.

### 2.3 Outcomes

The medical records including surgery reports of the cases were reviewed for details on location and severity of the disorder. Hypospadias was classified into three subgroups depending on the location of the meatus: anterior (glandular, coronal, and sub‐coronal), middle (distal penile, midshaft, and proximal penile), and posterior (penoscrotal, scrotal, and perineal) hypospadias. Our primary outcome was any type of hypospadias, irrespective of additional birth defects, while the three subtypes of hypospadias were used as secondary outcomes.

### 2.4 Statistical analysis

The maternal characteristics of cases and controls were listed as counts and percentages. Unadjusted odds ratios (OR) with 95% confidence intervals (CI) were estimated for the associations between the four primary exposures (maternal hypertensive disorders), as well as the hypertensive disorders stratified by exposure to antihypertensive medication, and hypospadias including its subtypes. Subsequently, we estimated adjusted odds ratios (aOR) using multivariable logistic regression analyses. Only variables that changed the effect estimate of the exposure of interest by more than 10% upon removal were retained as confounders in the final model. In case of fewer than three exposed cases or controls, only unadjusted ORs with 95% Fisher's exact 95% CI were estimated.

#### 2.4.1 Missing data

The numbers of missing data for all maternal characteristics are listed in Table 1. As almost all maternal characteristics and the exposure and outcome variables had limited numbers of missing data (<5%), no imputation was performed.

**Table 1:** Maternal characteristics and family history of hypospadias among hypospadias cases and unaffected controls

Characteristic	Controls (N = 1005) No. (%)	Hypospadias cases (N = 887) No. (%)
Maternal age at delivery		
18‐29 y	365 (36.3)	382 (43.1)
30‐34 y	452 (45.0)	346 (39.0)
≥35 y	182 (18.1)	155 (17.5)
Missing data	6 (0.6)	4 (0.5)
Race/ethnicity		
European Caucasian	973 (96.8)	862 (97.2)
Other	27 (2.7)	20 (2.3)
Missing data	5 (0.5)	5 (0.6)
Maternal education		
Low	101 (10.0)	114 (12.9)
Intermediate	529 (52.6)	454 (51.2)
High	370 (36.8)	316 (35.6)
Missing data	5 (0.5)	3 (0.3)
Parity		
0 previous births	457 (45.5)	579 (65.3)
≥1 previous births	548 (54.5)	308 (34.7)
Missing data	0 (0.0)	0 (0.0)
Pre‐pregnancy BMI (kg/m^2^)		
Underweight (<18.5)	39 (3.9)	35 (3.9)
Normal weight (18.5‐24.9)	666 (66.3)	574 (64.7)
Overweight (25.0‐29.9)	171 (17.0)	161 (18.2)
Obese (≥30.0)	59 (5.9)	70 (7.9)
Missing data	70 (7.3)	47 (5.3)
Fertility treatment		
Yes	60 (6.0)	63 (7.1)
No	944 (93.9)	820 (92.4)
Missing data	1 (0.1)	4 (0.5)
Periconceptional oral contraceptive use^a^		
Yes	245 (24.4)	186 (21.0)
No	751 (74.7)	695 (78.4)
Missing data	9 (0.9)	6 (0.7)
Folic acid use in recommended period^b^		
Yes, entire recommended period	176 (17.5)	177 (20.0)
Yes, only part of recommended period	386 (38.4)	302 (34.0)
No	369 (36.7)	346 (39.0)
Missing data	74 (7.4)	62 (7.0)
Periconceptional alcohol use^a^		
Yes	410 (40.8)	364 (41.0)
No	595 (59.2)	518 (58.4)
Missing data	0 (0.0)	5 (0.6)
Periconceptional smoking^a^		
Yes	227 (22.6)	195 (22.0)
No	778 (77.4)	688 (77.6)
Missing data	0 (0.0)	4 (0.5)
Family history of hypospadias		
Yes	6 (0.6)	60 (6.8)
No	999 (99.4)	827 (93.2)
Missing data	0 (0.0)	0 (0.0)

^a^ Any time from 3 mo preconception through the first 3 mo of pregnancy.

^b^ In the Netherlands, the recommended period for folic acid use is from 4 wk preconception through the first 10 wk of pregnancy.

#### 2.4.2 Sensitivity analyses

Three sensitivity analyses were performed using the analysis strategy outlined above. In the first analysis, we excluded cases and controls who had a first‐degree relative with hypospadias to minimise information bias due to familiarity with the disorder. Secondly, we restricted the study population to cases and controls born in the period 1990‐2011 with a known time lag between birth and completion of the questionnaire, in order to provide insight into the potential influence of the disparity in birth years between cases and controls. By stratifying the time lag into ≤5 years and >5 years, we tried to identify the role of misclassification due to memory effects. The third sensitivity analysis comprised the exclusion of cases and controls with a preterm birth to minimise the effect of early‐onset preeclampsia (<34 weeks of gestation), which usually results in preterm delivery.

The data were analysed using SPSS Statistics version 25 (IBM Corp).

### 2.5 Ethics approval

The initial data collection protocol for the AGORA data‐ and biobank and several updates were approved by the Regional Committee on Research Involving Human Subjects Arnhem‐Nijmegen.

## 3 RESULTS

We extracted 960 cases and 1050 male controls from the AGORA data‐ and biobank. Due to multiple gestations, 71 cases and 42 controls were excluded. Furthermore, we excluded two cases and three controls because of pre‐existing diabetes of the mother. As a result, 887 cases and 1005 controls were included in the analyses. Our patient population contained 366 new cases as well as 161 and 360 cases used in two previous studies.^6^, ^8^ Table 1 summarises the maternal characteristics of the cases and controls included. Patient mothers were younger at delivery and more likely to be primiparous and overweight or obese compared to control mothers. Mothers of controls slightly more often used oral contraceptives and folic acid supplements in the periconceptional period. The proportions of women who drank alcoholic beverages or smoked in that period were similar in the two groups. For cases, a family history of hypospadias was reported much more often than for control children.

In total, 150 (16.9%) patient mothers and 139 (13.8%) control mothers were diagnosed with one of the four hypertensive disorders of pregnancy. Table 2 shows the frequencies of the specific hypertensive disorders and the use of antihypertensive medication among the mothers of cases and controls. Among the latter, 15 (1.5%) women were diagnosed with chronic hypertension, 91 (9.1%) with gestational hypertension, 30 (3.0%) with preeclampsia, and 3 (0.3%) with preeclampsia superimposed on chronic hypertension. In the patient group, the numbers of diagnosed women were 16 (1.8%) for chronic hypertension, 87 (9.8%) for gestational hypertension, 45 (5.1%) for preeclampsia, and 2 (0.2%) for preeclampsia superimposed on chronic hypertension. Approximately 40% of the women with chronic hypertension were treated with antihypertensive medication during pregnancy, compared to only 13%‐24% of the women with gestational hypertension or preeclampsia.

**Table 2:** Associations between hypospadias and maternal hypertensive disorders stratified by treatment with antihypertensive medication during pregnancy

Exposure group	Controls (N = 1005) No. (%)	Cases (N = 887) No. (%)	Unadjusted OR (95% CI)	Adjusted OR (95% CI)^a^
No hypertension or use of antihypertensive medications	806 (80.2)	707 (79.7)	1.00 (Reference)	1.00 (Reference)
Any hypertension	139 (13.8)	150 (16.9)	1.23 (0.96, 1.58)	1.08 (0.83, 1.38)
Treated with antihypertensive medication	30 (3.0)	29 (3.3)	1.10 (0.66, 1.85)	0.92 (0.54, 1.57)
Not treated with antihypertensive medication	104 (10.3)	120 (13.5)	1.32 (0.99, 1.74)	1.15 (0.86, 1.53)
Chronic hypertension	15 (1.5)	16 (1.8)	1.22 (0.60, 2.48)	1.36 (0.66, 2.81)
Treated with antihypertensive medication	7 (0.7)	6 (0.7)	0.98 (0.33, 2.92)	1.09 (0.36, 3.32)
Not treated with antihypertensive medication	8 (0.8)	10 (1.1)	1.43 (0.56, 3.63)	1.60 (0.62, 4.15)
Gestational hypertension	91 (9.1)	87 (9.8)	1.09 (0.80, 1.49)	0.93 (0.68, 1.28)
Treated with antihypertensive medication	19 (1.9)	12 (1.4)	0.72 (0.35, 1.49)	0.57 (0.27, 1.19)
Not treated with antihypertensive medication	68 (6.8)	74 (8.3)	1.24 (0.88, 1.75)	1.07 (0.76, 1.53)
Preeclampsia	30 (3.0)	45 (5.1)	1.71 (1.07, 2.74)	1.37 (0.85, 2.22)
Treated with antihypertensive medication	4 (0.4)	11 (1.2)	3.14 (0.99, 9.89)	2.29 (0.72, 7.29)
Not treated with antihypertensive medication	25 (2.5)	34 (3.8)	1.55 (0.92, 2.62)	1.26 (0.74, 2.15)
Preeclampsia superimposed on chronic hypertension	3 (0.3)	2 (0.2)	0.76 (0.06, 6.66)^b^	–

^a^ OR adjusted for parity.

^b^ 95% Fisher's exact CI

Unadjusted and adjusted ORs for the associations between hypospadias and the four hypertensive disorders, stratified by antihypertensive medication exposure, are also provided in Table 2. All aORs were adjusted for parity, which proved to be the only true confounder in this study. Neither chronic nor gestational hypertension was associated with hypospadias, regardless of pharmacological treatment. Preeclampsia seemed to be weakly associated with hypospadias (aOR 1.37, 95% CI 0.85, 2.22). When treated with antihypertensive medication, the aOR for women with preeclampsia increased to 2.29 (95% CI: 0.72, 7.29), whereas the aOR for untreated pre‐eclamptic women was 1.26 (95% CI 0.74, 2.15). The time of onset was known for 80% of the women with preeclampsia. With 37.1% of the case mothers and 28.0% of the control mothers having early‐onset preeclampsia, the ORs for having a child with hypospadias were 2.12 (0.84, 5.34) and 1.39 (0.74, 2.62) for early and late onset, respectively. Because of small numbers, adjusted ORs could not be estimated for preeclampsia superimposed on chronic hypertension.

Table 3 provides the adjusted ORs for the associations between the four hypertensive disorders, stratified by antihypertensive medication exposure, and the three subtypes of hypospadias. Again, parity was the only confounder in these analyses. None of the hypertensive disorders of pregnancy were associated with anterior or middle hypospadias, except for an inverse association between treated gestational hypertension and anterior hypospadias (aOR 0.36, 95% CI 0.13, 0.99). Posterior hypospadias, in contrast, was associated with preeclampsia (aOR 3.09, 95% CI 1.49, 6.43). This association was apparent for both untreated preeclampsia (aOR 2.81, 95% CI 1.24, 6.38) and pharmacologically treated preeclampsia (aOR 4.96, 95% CI 1.08, 22.80). Of note, the latter OR was based on only three exposed cases, all with early‐onset preeclampsia, and four exposed controls.

**Table 3:** Associations between the three subtypes of hypospadias and maternal hypertensive disorders stratified by treatment with antihypertensive medication during pregnancy

Exposure group	Controls	Anterior hypospadias		Middle hypospadias		Posterior hypospadias	
	(N = 1005) No. (%)	(N = 579) No. (%)	aOR (95% CI)^a^	(N = 188) No. (%)	aOR (95% CI)^a^	(N = 99) No. (%)	aOR (95% CI)^a^
No hypertension or use of antihypertensive medications	806 (80.2)	469 (81.0)	1.00 (Reference)	150 (79.8)	1.00 (Reference)	70 (70.7)	1.00 (Reference)
Any hypertension	139 (13.8)	91 (15.7)	1.01 (0.76, 1.36)	34 (18.1)	1.13 (0.73, 1.69)	22 (22.2)	1.38 (0.81, 2.33)
Treated with antihypertensive medication	30 (3.0)	15 (2.6)	0.74 (0.39, 1.39)	8 (4.3)	1.20 (0.53, 2.69)	6 (6.1)	1.66 (0.66, 4.20)
Not treated with antihypertensive medication	104 (10.3)	75 (13.0)	1.12 (0.81, 1.55)	26 (13.8)	1.13 (0.71, 1.82)	16 (16.2)	1.36 (0.75, 2.46)
Chronic hypertension	15 (1.5)	12 (2.1)	1.56 (0.71, 3.39)	2 (1.1)	‐	2 (2.0)	‐
Treated with antihypertensive medication	7 (0.7)	5 (0.9)	1.23 (0.39, 3.89)	1 (0.5)	‐	0 (0.0)	‐
Not treated with antihypertensive medication	8 (0.8)	7 (1.2)	1.79 (0.64, 5.03)	1 (0.5)	‐	2 (2.0)	‐
Gestational hypertension	91 (9.1)	55 (9.5)	0.91 (0.63, 1.30)	22 (11.7)	1.10 (0.66, 1.82)	8 (8.1)	0.78 (0.36, 1.69)
Treated with antihypertensive medication	19 (1.9)	5 (0.9)	0.36 (0.13, 0.99)	4 (2.1)	0.90 (0.30, 2.70)	3 (3.0)	1.22 (0.35, 4.30)
Not treated with antihypertensive medication	68 (6.8)	49 (8.5)	1.10 (0.74, 1.63)	18 (9.6)	1.21 (0.70, 2.12)	5 (5.1)	0.68 (0.26, 1.76)
Preeclampsia	30 (3.0)	23 (4.0)	1.11 (0.63, 1.95)	9 (4.8)	1.27 (0.58, 2.76)	12 (12.1)	3.09 (1.49, 6.43)
Treated with antihypertensive medication	4 (0.4)	5 (0.9)	1.65 (0.44, 6.24)	3 (1.6)	2.77 (0.61, 12.61)	3 (3.0)	4.96 (1.08, 22.80)
Not treated with antihypertensive medication	25 (2.5)	18 (3.1)	1.05 (0.56, 1.95)	6 (3.2)	1.03 (0.41, 2.58)	9 (9.1)	2.81 (1.24, 6.38)
Preeclampsia superimposed on chronic hypertension	3 (0.3)	1 (0.2)	–	1 (0.5)	–	0 (0.0)	–

^a^ OR adjusted for parity.

In the first sensitivity analysis, we excluded 60 (6.8%) cases and 6 (0.6%) controls with a first‐degree family history of hypospadias, leaving 827 cases and 999 controls for analysis. The unadjusted and adjusted ORs differed only marginally from those in the primary analyses and are provided in Table S1 for all hypospadias subtypes combined. The results of the second sensitivity analysis for hypospadias as a group, after exclusion of 345 (38.9%) cases and 36 (3.6%) controls, are shown in Table S2. Only slight differences were seen in unadjusted and adjusted ORs compared to the primary analyses, except for treated preeclampsia with an aOR of 1.43 (0.35, 5.77) versus 2.29 (0.72, 7.29), respectively. After stratification on the time lag, quite different aORs were observed for completion of the questionnaire ≤5 years vs >5 years after delivery, but with wide 95% CIs. Clear differences were only detected for gestational hypertension, with aORs for the time lag ≤5 years being 0.29 (0.09, 0.90) and 2.05 (1.01, 4.19) for treated and untreated participants, respectively, as opposed to 0.57 (0.27, 1.19) and 1.07 (0.76, 1.53) in the primary analyses. Among the subtypes of hypospadias, only an aOR of 2.17 (1.02, 4.61) for anterior hypospadias associated with untreated gestational hypertension with a time lag ≤5 years stood out compared to the results in Table 3. In the third sensitivity analysis, in which we excluded 159 (17.9%) cases and 137 (13.6%) controls with a preterm birth, all results were comparable to the primary analyses, albeit with wider CIs. The aORs for posterior hypospadias and all preeclampsia (4.14 (1.70, 10.06)) as well as untreated preeclampsia (3.94 (1.54, 10.05)) were somewhat higher than in Table 3, while the aOR for pharmacologically treated preeclampsia could not be estimates due to small numbers.

## 4 COMMENT

### 4.1 Principal findings

In this population‐based case‐control study, we explored the associations between maternal hypertensive disorders and hypospadias in detail, by stratifying both exposures and outcomes. We observed clear associations between preeclampsia and posterior hypospadias for both treated and untreated preeclampsia. No other meaningful associations were found between maternal hypertension in general or specific hypertensive disorders and hypospadias or its subtypes.

### 4.2 Strengths of the study

A major strength of this study was the ability to study stratified exposures and outcomes by subtypes due to the use of a large and well‐established population‐based data collection. The detailed clinical data available in the AGORA data‐ and biobank, collected from medical files including surgical reports, assured proper diagnoses of hypospadias with clear differentiation between the hypospadias subtypes. The extensive questionnaires used during data collection enabled us to assess the presence or absence of the distinctive hypertensive disorders and the treatment with antihypertensive medication for each mother. In a validation study of the AGORA questionnaire used for prescription medication, the sensitivity for antihypertensive medication proved to be 0.83 while the specificity was 1.00.^28^ In addition, the questionnaire data were used to gather information on potential confounders, which are usually not available from medical records. The sensitivity analyses performed did not point towards large amounts of selection bias or information bias in the results from the primary analyses, despite the low participation rate among control parents.

### 4.3 Limitations of the data

The self‐reported nature of the exposure and confounder information may be a limitation of this study, especially since a portion of the mothers of both cases and controls completed the questionnaires several years after the index pregnancy. No clear differences were observed, however, between results based on completion of the questionnaire ≤5 years vs >5 years after delivery. The AGORA questionnaire was not validated for hypertensive disorders in pregnancy and comparable case‐control studies from the literature reported sensitivities of 46%‐100% for gestational hypertension and preeclampsia.^29^ Therefore, some misclassification of exposure seems likely but is presumably nondifferential, since case mothers would not have associated these hypertensive disorders with hypospadias in their sons. The same may apply to the lifestyle factors included in the study, as these were quite equally distributed among cases and controls and did not play a role as confounders. If differential recall would have occurred in any of these factors, however, confounding could have been obscured. Still, we believe our main results to be valid, especially since preeclampsia is a clearly defined condition that is not easily misclassified, and neither is the confounder parity. The prevalence rates of preeclampsia in this study were also in the same order of magnitude as in two very large studies.^23^, ^26^ Due to small numbers and lack of information, we were not able to stratify preeclampsia by severity, estimate the associations between preeclampsia superimposed on chronic hypertension and hypospadias, use the information on timing and type of medication, or fully examine the potential confounders in the stratified analyses. For some of the same reasons, we could not estimate the associations between chronic hypertension and specific subtypes of hypospadias.

### 4.4 Interpretation

In the study by Brouwers et al,^6^ with 161 of the 305 hypospadias cases overlapping with the current study, preeclampsia was found to be strongly associated with posterior hypospadias, while the ORs for preeclampsia and anterior and middle hypospadias were elevated as well, but with CIs including unity. This was the case for all three subtypes of hypospadias in the study by van Rooij et al,^8^ with 360 of the 405 cases overlapping with ours. However, these studies made no distinction between treated and untreated preeclampsia. When we made this distinction in the current study, the OR for middle hypospadias seemed to be elevated for treated preeclampsia only, but with a wide CI, whereas clearly increased risks of posterior hypospadias were observed for both pharmacologically treated and untreated preeclampsia. In one of the few studies that reported using mutually exclusive groups of hypertensive disorders, van Gelder et al^23^ found an increased risk of second and third‐degree hypospadias among women with untreated preeclampsia only in the United States. They also found an increased risk of second‐ and third‐degree hypospadias for untreated gestational hypertension,^23^ which we only observed among participants who completed the questionnaire ≤5 years after delivery, but especially for anterior hypospadias. We could not replicate the increased risks of second‐ and third‐degree hypospadias for untreated hypertension in general found by others in the United States.^18^, ^21^, ^22^ Agopian et al observed similar risks for hypertension and gestational hypertension, not taking treatment into account.^25^ Several others reported associations between gestational hypertension and hypospadias in general,^6^, ^16^, ^19^, ^20^, ^23^, ^25^ whereas our study and the cohort study of Arendt et al in Denmark that also used mutually exclusive groups of hypertensive disorders, showed no associations.^26^ Similar to two previous studies,^16^, ^25^ we did not find associations between chronic hypertension and hypospadias either, but two other larger studies did report this association.^23^, ^26^ Preeclampsia was clearly associated with hypospadias in general in several studies,^6^, ^8^, ^15^, ^17^, ^20^, ^23^, ^24^, ^26^ whereas we observed only a weak association between preeclampsia and hypospadias as a group. These variations in findings may partly be due to differences in the definitions and demarcations of the hypertensive disorders, in the mix of subtypes of hypospadias, and in treatment regimens among the various study populations. Timing of onset of preeclampsia may also play a role, as our results point towards an increased risk of hypospadias associated with early‐onset and probably more severe preeclampsia.

The results of this study cannot be explained by a contributing role for diabetes as was recently found in a study on several other birth defects.^30^ Women with pre‐existing diabetes were excluded from the study, and only a limited number of case mothers (n = 4) and control mothers (n = 5) reported gestational diabetes in the aetiologically relevant period. Other birth defects (cryptorchidism and kidney defects) were only observed in two of the 12 cases with posterior hypospadias and a mother with preeclampsia. Our results for preeclampsia underline the hypothesis of aetiological heterogeneity among the subtypes of hypospadias that was suggested in our previous studies, in which primiparity, multiple pregnancy, preterm birth, low birthweight, and being small for gestational age were also associated with posterior hypospadias. As the male external genitalia develop between the 8th and 14th weeks of gestation, however, the development of hypospadias cannot be influenced by factors that occur later in pregnancy, such as preeclampsia and treatment with antihypertensive medication. Therefore, a shared risk factor, that is placental dysfunction in early pregnancy, may be the explanation for the associations between preeclampsia and the other above‐mentioned factors and posterior hypospadias.^6^, ^8^ During development of the male genitalia, the placental production of human chorionic gonadotropin (HCG) is crucial, as HCG is responsible for regulating the production of testosterone in the Leydig cells.^31^, ^32^, ^33^, ^34^ Insufficient production of HCG due to placental dysfunction could lead to low testosterone levels resulting in abnormal development of the male external genitalia.

## 5 CONCLUSIONS

We observed that both pharmacologically treated and untreated preeclampsia were associated with posterior hypospadias, with a higher risk estimate for treated early‐onset preeclampsia. Our results underscore the hypothesis of aetiological heterogeneity among the subtypes of hypospadias, in which severe forms of hypospadias are associated with pregnancy‐related risk factors. Our findings also suggest that preeclampsia and posterior hypospadias may have a shared risk factor in placental insufficiency.

## Supporting information

Table S1‐S2
