# Supplementary material for: Maternal hypertensive disorders and subtypes of hypospadias: A Dutch case‐control study
Source: Paediatr Perinat Epidemiol. 2020 Jul 13;34(6):687–95. doi: 10.1111/ppe.12683 (PMC7689729; doi:10.1111/ppe.12683)
Supplement: Supplementary file 1 — Table S1‐S2 [file PPE-34-687-s001.docx]

eTable 1. Associations between hypospadias and maternal hypertensive disorders stratified by

treatment with antihypertensive medication during pregnancy for hypospadias cases and controls

without family history of hypospadias.

| **Exposure group** | **Controls**  **(N=999)**  **No. (%)** | **Cases**  **(N=827)**  **No. (%)** | **Unadjusted OR (95% CI)** | **Adjusted OR (95% CI)^a^** |
| --- | --- | --- | --- | --- |
| No hypertension or use of antihypertensive medications | 803 (80.1) | 659 (81.2) | 1.00 (Reference) | 1.00 (Reference) |
| Any hypertension | 138 (13.8) | 139 (16.8) | 1.23 (0.95, 1.59) | 1.07 (0.82, 1.39) |
| Treated with antihypertensive medication | 29 (2.9) | 27 (3.3) | 1.13 (0.67, 1.94) | 0.94 (0.55, 1.62) |
| Not treated with antihypertensive medication | 104 (10.4) | 111 (13.4) | 1.30 (0.98, 1.73) | 1.14 (0.85, 1.53) |
| Chronic hypertension | 15 (1.5) | 15 (1.8) | 1.22 (0.59, 2.51) | 1.36 (0.65, 2.84) |
| Treated with antihypertensive medication | 7 (0.7) | 5 (0.6) | 0.81 (0.28, 2.76) | 0.94 (0.29, 3.06) |
| Not treated with antihypertensive medication | 8 (0.8) | 10 (1.2) | 1.52 (0.60, 3.88) | 1.73 (0.67, 4.48) |
| Gestational hypertension | 90 (9.0) | 80 (9.7) | 1.08 (0.79, 1.49) | 0.93 (0.67, 1.29) |
| Treated with antihypertensive medication | 18 (1.8) | 12 (1.5) | 0.81 (0.39, 1.70) | 0.64 (0.30, 1.36) |
| Not treated with antihypertensive medication | 68 (6.8) | 67 (8.1) | 1.20 (0.84, 1.71) | 1.05 (0.73, 1.50) |
| Preeclampsia | 30 (3.0) | 42 (5.1) | 1.71 (1.06, 2.76) | 1.37 (0.84, 2.23) |
| Treated with antihypertensive medication | 4 (0.4) | 10 (1.2) | 3.05 (0.95, 9.76) | 2.20 (0.69, 7.16) |
| Not treated with antihypertensive medication | 25 (2.5) | 32 (3.9) | 1.56 (0.92, 2.66) | 1.27 (0.74, 2.18) |
| Preeclampsia superimposed hypertension | 3 (0.3) | 2 (0.2) | 0.80 (0.07, 7.05)^b^ | - |

^a^ OR adjusted for parity.

^b^95% Fisher's Exact CI

eTable 2. Associations between hypospadias and maternal hypertensive disorders stratified by treatment

with antihypertensive medication during pregnancy for hypospadias cases and controls born in the period

1990-2011 with a known time lag between birth and completion of the AGORA questionnaire.

| **Exposure group** | **Controls**  **(N=969)**  **No. (%)** | **Cases**  **(N=542)**  **No. (%)** | **Unadjusted OR (95% CI)** | **Adjusted OR**  **(95% CI)^a^** | **Adjusted OR**  **(95% CI)^a^ with time lag ≤5 years** | **Adjusted OR**  **(95% CI)^a^ with time lag >5 years** |
| --- | --- | --- | --- | --- | --- | --- |
| No hypertension or use of antihypertensive medications | 776 (80.1) | 440 (81.2) | 1.00 (Reference) | 1.00 (Reference) | 1.00 (Reference) | 1.00 (Reference) |
| Any hypertension | 137 (14.1) | 87 (16.1) | 1.12 (0.84, 1.50) | 1.02 (0.76, 1.38) | 1.15 (0.72, 1.81) | 0.90 (0.59, 1.36) |
| Treated with antihypertensive medication | 29 (3.0) | 15 (2.8) | 0.91 (0.48, 1.72) | 0.84 (0.44, 1.59) | 0.61 (0.27, 1.37) | 0.87 (0.30, 2.49) |
| Not treated with antihypertensive medication | 103 (10.6) | 72 (13.3) | 1.23 (0.89, 1.70) | 1.12 (0.81, 1.55) | 1.48 (0.86, 2.55) | 0.96 (0.62, 1.50) |
| Chronic hypertension | 15 (1.6) | 10 (1.9) | 1.18 (0.52, 2.64) | 1.23 (0.55, 2.77) | 1.22 (0.40, 3.70) | 0.89 (0.24, 3.36) |
| Treated with antihypertensive medication | 7 (0.7) | 4 (0.7) | 1.01 (0.29, 3.46) | 1.09 (0.32, 3.77) | 1.43 (0.32, 6.49) | - |
| Not treated with antihypertensive medication | 8 (0.8) | 6 (1.1) | 1.32 (0.46, 3.85) | 1.35 (0.46, 3.93) | 1.01 (0.20, 5.08) | 1.59 (0.37, 6.76) |
| Gestational hypertension | 89 (9.4) | 51 (9.6) | 1.01 (0.70, 1.45) | 0.91 (0.63, 1.32) | 1.13 (0.64, 2.00) | 0.75 (0.44, 1.26) |
| Treated with antihypertensive medication | 18 (1.9) | 7 (1.3) | 0.69 (0.28, 1.66) | 0.61 (0.25, 1.48) | 0.29 (0.09, 0.90) | 1.35 (0.32, 5.75) |
| Not treated with antihypertensive medication | 67 (6.9) | 44 (8.1) | 1.16 (0.78, 1.72) | 1.05 (0.70, 1.57) | 2.05 (1.01, 4.19) | 0.74 (0.42, 1.30) |
| Preeclampsia | 30 (3.2) | 24 (4.5) | 1.14 (0.82, 2.44) | 1.24 (0.71, 2.16) | 1.13 (0.46, 2.81) | 1.36 (0.66, 2.79) |
| Treated with antihypertensive medication | 4 (0.4) | 4 (0.7) | 1.76 (0.44, 7.09) | 1.43 (0.35, 5.77) | - | 1.01 (0.18, 5.59) |
| Not treated with antihypertensive medication | 25 (2.6) | 20 (3.7) | 1.41 (0.78, 2.57) | 1.25 (0.68, 2.29) | 0.94 (0.36, 2.42) | 1.53 (0.69, 3.37) |
| Preeclampsia superimposed on chronic hypertension | 3 (0.3) | 2 (0.4) | 1.18 (0.10, 10.30)^b^ | - | - | - |

^a^ OR adjusted for parity.

^b^ 95% Fisher's Exact CI
